# Posterior Belly of the Digastric Muscle as a Reliable and Consistent Landmark for the Identification of the Facial Nerve Trunk During Parotidectomy

**DOI:** 10.7759/cureus.80413

**Published:** 2025-03-11

**Authors:** Arun Singh, Shivani B Paruthy, Vaibhav Kuraria, Mohit Dhawaria, Sunil Kumar Singh, Dhananjay Khera, Abhinav Kumar, Singamsetty S Madhuri, Sonali Chaudhary, Hinduja Raju

**Affiliations:** 1 General Surgery, Vardhman Mahavir Medical College and Safdarjung Hospital, New Delhi, IND; 2 General Surgery, Maulana Azad Medical College, New Delhi, IND; 3 Surgical Oncolgy, Vardhman Mahavir Medical College and Safdarjung Hospital, New Delhi, IND; 4 Psychiatry, Vardhman Mahavir Medical College and Safdarjung Hospital, New Delhi, IND

**Keywords:** facial nerve dissection, facial nerve surgery, pleomorphic adenomas, posterior belly of the digastric muscle, superficial parotidectomy, tragal pointer

## Abstract

Background

Identification and preservation of the facial nerve trunk (FNT) are critical during parotidectomy to prevent complications such as facial paralysis. Due to its anatomical proximity and accessibility, the posterior belly of the digastric muscle (PBDM) has emerged as a consistent and reliable intraoperative landmark for localizing the FNT. This study aims to review the experience of a single institution in superficial parotidectomy, with a focus on identifying the FNT using the PBDM.

Methods

This retrospective observational study reviewed 24 cases of parotidectomy performed at Safdarjung Hospital, New Delhi, from January 2021 to January 2024. Inclusion criteria included patients with parotid tumors, excluding those with preoperative facial nerve palsy or prior head and neck radiation or surgery. Key anatomical landmarks, such as the PBDM and tragal pointer, were used to identify the FNT.

Results

The PBDM was consistently identified as a reliable landmark. The FNT was located approximately 15-20 mm (17 ± 0.87 mm) superior and medial to the insertion of the PBDM. The distance from the PBDM to the FNT in this study was significantly greater compared to previous studies (p < 0.001), reinforcing its reliability as a consistent anatomical landmark. No cases of facial nerve paresis were observed.

Conclusion

The PBDM is a reliable and consistent landmark for FNT identification, minimizing the risk of facial nerve injury. This approach is valuable for optimizing outcomes in parotid surgery.

## Introduction

The parotid gland, a major salivary gland located in the lateral face over the ramus of the mandible, is a complex anatomical structure that presents significant challenges during surgical procedures. The majority (80%-85%) of parotid tumors are benign, with pleomorphic adenoma being the most common, accounting for 60%-70% of cases [1]. Superficial parotidectomy involves the dissection of the facial nerve and the removal of the parotid gland surrounding it. This procedure is one of the most commonly performed surgeries in head and neck surgery. During superficial parotidectomy, the facial nerve trunk (FNT) can be exposed through an anterograde or retrograde approach. The identification and careful preservation of the FNT are critical to avoid postoperative complications, including facial nerve paralysis [1]. 

Facial nerve injury is the most common complication of parotid surgery due to the close relationship between the nerve and the gland. Although the anatomical theory is debated, it is traditionally believed that the facial nerve divides the parotid gland into deep and superficial lobes. Between these two lobes, a potential plane is formed by the facial nerve and the vessels surrounding it. If the surgeon fails to identify the FNT and navigate along the nerve and its branches, dissection within this plane becomes extremely challenging [2].

Since Janes first described FNT exposure using the anterograde method in 1940 [3], multiple landmarks have been established for identifying the FNT. The tragal pointer, once considered a pointer to identify FNT, is unreliable intraoperatively, as the nerve lies 1.5-2 cm deep and inferior to it [4]. Additionally, its cartilaginous structure makes it variable and difficult to use during surgery.

Bony landmarks are more consistent. Brintnall et al. identified the relationship between the FNT and the tympanomastoid fissure (TMF) [5]. However, because of its deep-seated nature and the need for extensive periosteal striping, which is often excessive in superficial parotidectomy, this landmark is not widely used.

The digastric muscle, located in the anterior neck, consists of two distinct bellies: the anterior and posterior bellies. Numerous studies have explored its complex anatomy and clinical significance in various surgical procedures [6,7]. The posterior belly of the digastric muscle (PBDM) is known to traverse the posteromedial surface of the parotid gland, often near the FNT, making it a valuable anatomical reference point for the surgeon [8].

This study aims to review the experience of a single institution in superficial parotidectomy, with a focus on identifying the FNT using the PBDM.

## Materials and methods

Aim of the study

This study aims to determine the reliability and consistency of the PBDM as a landmark for FNT identification during parotidectomy.

Study design and patient selection

This retrospective study included 24 patients with pleomorphic adenoma who underwent superficial parotidectomy at Vardhman Mahavir Medical College and Safdarjung Hospital, New Delhi, India, between January 1, 2021, and January 31, 2024. We reviewed the patients' electronic medical records. This study was approved by the Institutional Ethics Committee (ref no. IEC/VMMC/SJH/Cert/04-2024/39). The inclusion criteria were as follows: all diagnosed cases of pleomorphic adenoma of the parotid gland with superficial lobe involvement. The exclusion criteria included preoperative facial nerve involvement, deep lobe involvement, and re-do surgery.

All procedures were performed under general anesthesia, with the neck slightly extended and the head rotated to the contralateral side. A modified Blair incision was made, and the wound was opened in layers to raise the flaps. The insertion of the sternocleidomastoid and the PBDM were identified, with the PBDM traced anteriorly. The FNT was located, and the tragal pointer was identified. Measurements were taken from the PBDM and FNT, as well as the tragal pointer to the FNT. Dissection continued along the FNT, and superficial parotidectomy was performed while preserving all branches of the facial nerve. The wound was closed in layers.

The distance from each landmark (tragal pointer and PBDM) to the FNT was measured using vernier calipers. Standard operative notes for superficial parotidectomy were reviewed, including pre- and postoperative diagnoses, type of surgical procedure, intraoperative findings (tumor size, lobe involved, distances from tragal pointer and PBDM to FNT), FNT involvement, intraoperative time, blood loss, and patient recovery.

Following data collection from the medical records department, the intraoperative notes were thoroughly reviewed, and the standard procedure for superficial parotidectomy was analyzed.

Data collection and statistical analyses

The collected data (using MS Excel, Microsoft Corp., Redmond, WA, United States) were analyzed using IBM SPSS Statistics for Windows, Version 29.0.2.0 (Released 2023; IBM Corp., Armonk, NY, United States). Continuous variables, such as the distance between anatomical landmarks and the FNT, were summarized using descriptive statistics, including means, standard deviations (SDs), ranges, and confidence intervals (CIs). Categorical variables, such as demographic data, were presented as frequencies and percentages.

To assess variability, the coefficient of variation for the measurements was calculated. A one-sample t-test was used to determine whether the mean distances from the PBDM and tragal pointer to the FNT significantly deviated from established reference values in similar studies.

## Results

**Figure 1:**
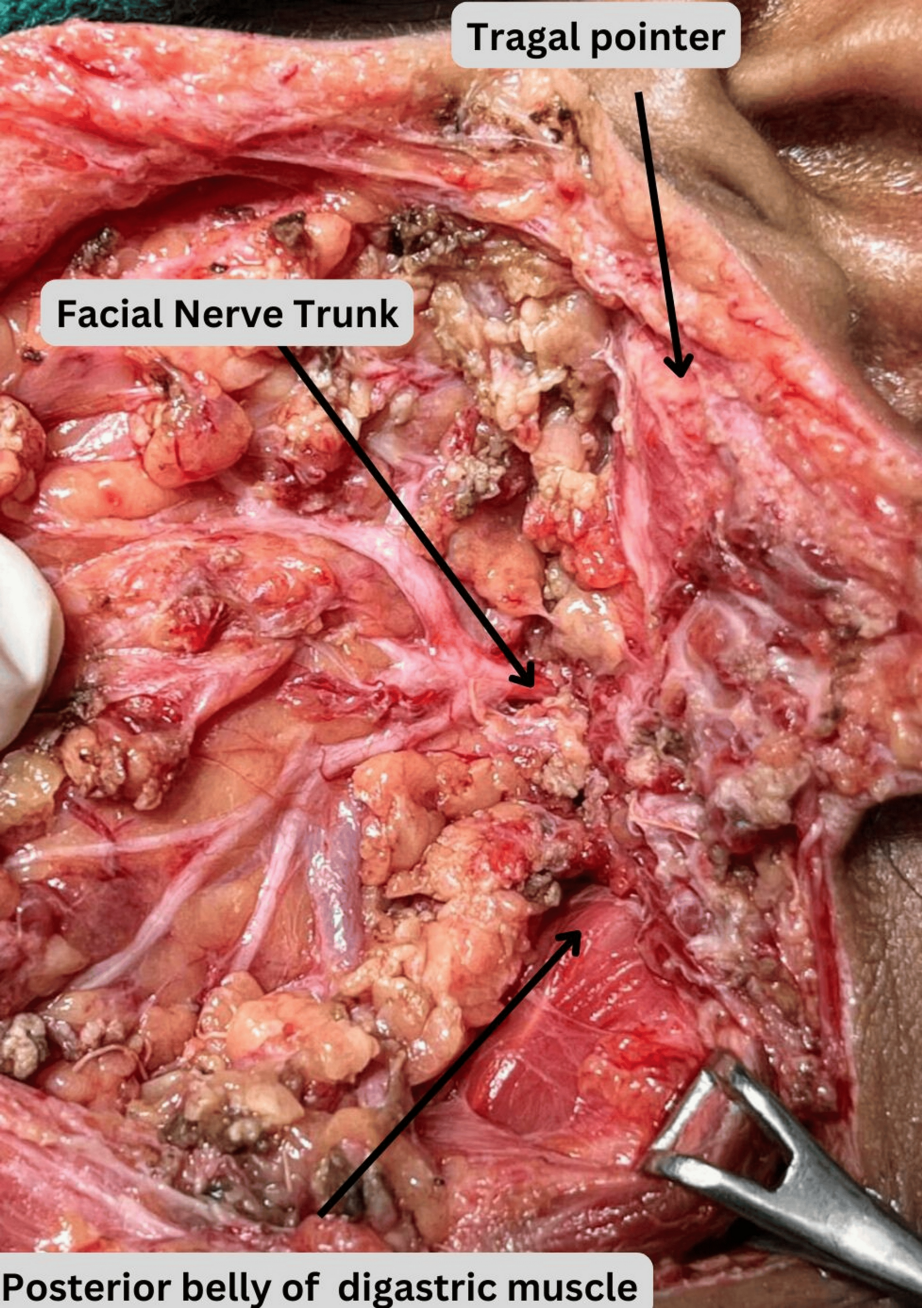
Intraoperative image of superficial parotidectomy

The study included 24 patients (14 male and 10 female participants), resulting in a male-to-female ratio of 1.4:1. The mean age of participants was 42.5 years (range, 28-58 years). The mean BMI was 23.78 kg/m^2^ (range, 22-24.9 kg/m^2^). The mean distance from the PBDM to the FNT was 17 mm, with a range of 15-20 mm and a standard deviation of 0.87 mm, indicating low variability among patients. In contrast, the distance from the tragal pointer to the FNT had a mean of 23.5 mm, with a range of 5-43 mm and a standard deviation of 11.96 mm, reflecting substantial variability among patients (Figure 1, Table 1).

**Table 1:** Comparison of distances from the FNT to the tragal pointer and PBDM FNT: facial nerve trunk, PBDM: posterior belly of the digastric muscle.

Patients	PBDM (in mm)	Tragal pointer (in mm)
1	16	7
2	15	5
3	17	8
4	17	6
5	16	8
6	16	6
7	17	7
8	17	23
9	17	22
10	17	34
11	20	27
12	17	37
13	17	20
14	16	34
15	17	32
16	18	24
17	17	40
18	17	43
19	17	34
20	16	18
21	17	26
22	17	24
23	17	27
24	18	28

A one-sample t-test was conducted to compare the distances from the PBDM and tragal pointer to the FNT in the present study with values reported in prior studies. The results are summarized in Table 2.

**Table 2:** Comparison of the distances from the PBDM and tragal pointer to the FNT in the present study and in prior studies PBDM: posterior belly of the digastric muscle. *Significant: p-value < 0.001.

Study	Landmark	Mean distance (±SD, mm)	Comparison (t-statistic)	p-value
Present study	PBDM	17 ± 0.87	-	-
Saha et al. [2]	PBDM	8.03 ± 1.59	59.61	<0.001*
Pather and Osman [9]	PBDM	14.6 ± 4.7	14.68	<0.001*
de Ru et al. [10]	PBDM	4.8 ± 2.34	78.08	<0.001*
Present study	Tragal pointer	23.5 ± 11.96	-	-
Saha et al. [2]	Tragal pointer	16.36 ± 1.91	1.41	0.17
Pather and Osman [9]	Tragal pointer	34 ± 6.7	-5.63	<0.001*
de Ru et al. [10]	Tragal pointer	8 ± 3.6	5.83	<0.001*

The PBDM showed significantly greater distances to the FNT in the present study compared to all prior studies (p < 0.001), reinforcing its reliability as a consistent anatomical landmark.

The tragal pointer exhibited significant variability across studies. The distance in the present study was significantly lower than that reported by Pather and Osman [9] (p < 0.001) but higher than that of de Ru et al. [10] (p < 0.001). No significant difference was found when compared to Saha et al. [2] (p = 0.17).

No permanent facial nerve injuries were reported.

## Discussion

Accurate identification of the FNT is crucial during parotidectomy to minimize complications such as nerve injury and postoperative facial paresis. Although several anatomical landmarks have been described for localizing the FNT, each has its advantages and limitations. The most commonly referenced landmarks include the TMF and the PBDM (Table 3) [9-13].

**Table 3:** Comparison of the present study with similar studies published in the literature FNT: facial nerve trunk, PBDM: posterior belly of the digastric muscle.

Study	Distance between the FNT and PBDM (mm)	Mean (±SD)
Saha et al. [2]	6–11.5	8.03 ± 1.59 mm
Pather and Osman [9]	9.7–24.3	14.6 ± 4.7 mm
de Ru et al. [10]	4.5–9	4.8 ± 2.34 mm
Present study	15–20	17 ± 0.87 mm

The findings of this study provide strong evidence supporting the reliability of the PBDM as a consistent landmark for FNT identification during parotidectomy. In the present study, the mean distance from the PBDM to the FNT was 17 mm, with low variability (SD = 0.87 mm), demonstrating high consistency. Comparisons with prior studies revealed that the mean distance in our study was significantly greater than that reported by Pather and Osman [9] (14.6 mm, p < 0.001), de Ru et al. [10] (4.8 mm, p < 0.001), and Saha et al. [2] (8.03 mm, p < 0.001) (Table 2), highlighting PBDM's robustness as an anatomical landmark across different populations.

In contrast, the tragal pointer showed considerable variability in its distance from the FNT, with a mean of 23.5 mm (SD = 11.96 mm) in this study. Significant differences were observed compared to the findings of Pather and Osman [9] (34 mm, p < 0.001) and de Ru et al. [10] (8 mm, p < 0.001). However, no significant difference was found when compared to the study by Saha et al. [2] (16.36 mm, p = 0.17) (Table 2). This variability underscores the limitations of the tragal pointer as a reliable landmark, influenced by its anatomical mobility and structural variability.

Although frequently cited, the TMF presents particular challenges for intraoperative use. Identifying the TMF often necessitates periosteal stripping, which involves navigating through parotid tissue and can be obscured by the sternocleidomastoid tendon. Furthermore, studies have reported variability in the distance between the TMF and the FNT. de Ru et al. documented a distance of 2-3 mm [10], while Pather and Osman reported a broader range of 4.9-18.6 mm [9], underscoring its inconsistent reliability.

The PBDM, a muscular landmark, has gained prominence due to its ease of identification and consistent anatomical relationship with the FNT. Pather and Osman noted that the PBDM’s position may vary based on individual factors such as race and sex [9]. Additionally, the tragal pointer, another landmark for FNT identification, exhibits significant variability due to its cartilaginous nature, intraoperative mobility during traction of ear lobule, and asymmetrical and blunt tip. Studies by Rea et al. [11] and Pather and Osman [9] reported distances of 6.9 ± 1.8 mm and 24.3-49.2 mm, respectively, diminishing its reliability as a consistent landmark.

Additional landmarks, such as the stylomastoid artery and the postauricular artery, have been suggested to aid in locating the FNT. Although the stylomastoid artery is occasionally visible intraoperatively, its inconsistent anatomical variations limit its reliability as a landmark [13,14]. Similarly, the postauricular artery, being small and embedded within dense subcutaneous tissue, poses challenges for routine surgical use [14].

Recent studies have explored various anatomical landmarks to aid in the identification of the FNT during parotidectomy. A systematic review by Ji et al. analyzed distances from common landmarks to the FNT, reporting mean distances of 13.6 mm (from six studies) for the tragal pointer and 8.79 mm (from seven studies) for the PBDM [15]. The study concluded that using multiple landmarks in conjunction is essential to reducing the risk of facial nerve damage during surgery [15].

Furthermore, Micucci et al. highlighted the parotid-mastoid fascia as an additional landmark for FNT identification [16]. They suggested that the parotid-mastoid segment, particularly the portion overlying the FNT, could be utilized in conjunction with other standard anatomical landmarks to help identify the FNT intraoperatively [16].

Superficial parotidectomy is one the most common procedures performed in head and neck surgery. For trainees and junior surgeons seeking to improve speed and accuracy, the identification of critical landmarks for the FNT is essential. This study offers objective data and a reliable method through a comparative analysis with existing literature. Additionally, it offers practical surgical guidance by focusing on easily identifiable key landmarks that do not require extensive dissection, thus improving safety and efficiency in surgical procedures. Furthermore, the findings help reduce variability in intraoperative techniques and offer valuable insights for both experienced and trainee surgeons.

Limitations

The study was conducted on only 24 patients, which may limit the overall generalizability of the findings. Larger studies involving more participants could provide more robust evidence. As the study was conducted at a single institution (Safdarjung Hospital, New Delhi), the results may not fully represent diverse populations or different surgical settings. Retrospective studies rely on previously recorded data, which may introduce biases, such as incomplete or inconsistent documentation. Although comparisons were made with other studies, variability in study methodologies (e.g., patient demographics, surgical techniques, or measurement tools) could affect the applicability of the findings.

## Conclusions

Parotidectomy requires a thorough understanding of the facial nerve and its course. Accurate localization of the FNT is critical to minimizing injury and complications during the procedure. Among the anatomical landmarks studied, the PBDM proved to be the most reliable and consistent landmark for FNT identification. In contrast, the tragal pointer showed significant variability, limiting its standalone utility. Although the TMF and vascular landmarks offer supplementary guidance, their inconsistent anatomy, challenging localization, and risk of nerve injury during identification pose notable difficulties. This study confirms that the PBDM is a consistent and reliable landmark for the identification of the FNT during parotidectomy.
